# Claudin 18.2 Positivity Thresholds and Candidate Populations for Targeted Therapy in Pancreatic Ductal Adenocarcinoma: A Real-World Retrospective Cohort

**DOI:** 10.3390/cancers18172827

**Published:** 2026-09-01

**Authors:** Güner Akgüner, Elif Haznedaroğlu Benlioğlu, Özgen Ahmet Yıldırım, Ayşegül İlhan Güleşen, Batuhan Günel, Ata Türker Arıkök, Ömür Berna Çakmak Öksüzoğlu, Kadriye Bir Yücel

**Affiliations:** 1 Department of Medical Oncology, Ankara Etlik City Hospital, Ankara 06170, Türkiye; elifhaznedaroglu9@gmail.com (E.H.B.); ozgenayildirim@gmail.com (Ö.A.Y.); ayse_ilhan85@hotmail.com (A.İ.G.); bernaoksuzoglu@yahoo.com (Ö.B.Ç.Ö.); kadriyebiryucel@gmail.com (K.B.Y.); 2 Department of Pathology, Ankara Etlik City Hospital, Ankara 06170, Türkiye; batuhangunel25@gmail.com (B.G.); atalina2005@yahoo.com (A.T.A.)

**Keywords:** claudin 18.2, pancreatic ductal adenocarcinoma, targeted therapy, treatment eligibility

## Abstract

Background/Objectives: Claudin 18.2 (CLDN18.2) is expressed in a substantial proportion of pancreatic ductal adenocarcinoma (PDAC) and has emerged as a promising therapeutic target. However, the positivity threshold for enrollment in CLDN18.2-directed trials remains undefined, and ongoing studies use different criteria. We quantified how different CLDN18.2 thresholds influence the size of the candidate population within the same PDAC cohort. Methods: In this retrospective single-center study, CLDN18.2 was assessed by immunohistochemistry (43-14A clone, Ventana BenchMark) in 99 PDAC patients (metastatic, *n* = 71; non-metastatic, *n* = 28). The candidate population was evaluated at three thresholds: two used in current CLDN18.2 trials (any-intensity [1+/2+/3+] staining in ≥40% and moderate-to-strong [2+/3+] staining in ≥75% of tumor cells) and an exploratory lower bound of any detectable expression (≥5%). Between-threshold reclassification was analyzed as paired data; clinicopathological and survival analyses were exploratory. Results: The candidate proportion was 40.4% (40/99) at ≥75%, 43.4% (43/99) at ≥40%, and 64.6% (64/99) at ≥5%; among metastatic patients, it was 36.6%, 38.0%, and 59.2%, respectively. Lowering the threshold from ≥75% to ≥40% caused virtually no reclassification (1/71 metastatic, 1.4%; McNemar *p* = 1.0), whereas lowering it to ≥5% reclassified 16 metastatic patients (22.5%; 95% CI, 14.4–33.5; *p* < 0.001) as candidates. Among the 64 tumors with detectable expression, median staining was 80% (IQR, 20–90%). Survival analyses were exploratory and underpowered (52 deaths; 80% power for HR ≥ 2.24); no association between CLDN18.2 and overall or progression-free survival was detected at any threshold. Conclusions: The stringent thresholds used in current drug development identified nearly identical candidate populations, whereas relaxation to any detectable expression substantially enlarged the pool; CLDN18.2 behaved as a targetable rather than a prognostic biomarker. Our findings highlight the need for a PDAC-specific, response-based CLDN18.2 threshold.

## Simple Summary

Pancreatic ductal adenocarcinoma (PDAC) is among the deadliest cancers, and new treatment targets are urgently needed. Claudin 18.2 is a protein normally confined to the stomach that can also appear on pancreatic cancer cells, and several drugs against it are now being tested. Which patients are considered candidates for these drugs depends on a cut-off: how much of the tumor must carry the protein. In gastric cancer, this cut-off is at least 75% of tumor cells, but the right cut-off for pancreatic cancer has not been established, and different trials use different ones. Using the same validated test employed in gastric cancer trials, we measured Claudin 18.2 in 99 patients and asked how much the choice of cut-off changes the number who would be considered candidates. Defining the right cut-off for Claudin 18.2 in pancreatic cancer will be essential to identify the patients who could benefit from these emerging treatments.

## 1. Introduction

Pancreatic ductal adenocarcinoma (PDAC) is among the most lethal solid tumors, with a five-year survival below 13%; most patients present with locally advanced or metastatic disease, when a cure is no longer attainable [1,2]. Metastatic disease is biologically aggressive, and its course is short: despite multi-agent cytotoxic regimens such as FOLFIRINOX and gemcitabine–nab-paclitaxel, median overall survival remains below one year, and options beyond the first line are limited [3,4]. The persistence of this poor prognosis, largely unchanged by conventional chemotherapy, has intensified the search for actionable targets in a disease where most recurrent molecular alterations are still not druggable [5]. Following the promising results of daraxonrasib in RAS-targeted therapy [6], the therapeutic potential of CLDN18.2-targeted approaches in PDAC remains an area of considerable interest.

Claudin 18.2 (CLDN18.2), a tight-junction protein physiologically restricted to gastric epithelium, is ectopically activated in PDAC and its precursor lesions and has emerged as one such target [7,8]. In gastric and gastroesophageal junction cancer, the anti-CLDN18.2 antibody zolbetuximab improved survival in two phase 3 trials and reached approval [9,10]; in PDAC, anti-CLDN18.2 targeted therapy options are advancing through early-phase testing [11,12]. In non-metastatic PDAC, where neoadjuvant and adjuvant strategies built on systemic chemotherapy and radiotherapy are central to management, CLDN18.2-directed approaches are also beginning to be explored: a registered phase II trial, not yet recruiting, aims to evaluate a CLDN18.2 antibody–drug conjugate combined with chemotherapy as neoadjuvant therapy in CLDN18.2-positive borderline-resectable PDAC, followed by surgery [13]. Although neoadjuvant chemotherapy has been reported to alter CLDN18.2 expression in different directions—increased following gemcitabine-based treatment in experimental models [14] and reduced after neoadjuvant chemotherapy in a real-world series [15]—the clinical implications of this modulation remain undefined.

Access through that route, however, is controlled by the positivity threshold, and in PDAC this threshold has never been standardized. The zolbetuximab companion diagnostic requires moderate-to-strong membranous staining in at least 75% of tumor cells [9,10], but that cut-off was calibrated in gastric cancer, and its transferability to PDAC is unproven. Ongoing PDAC trials compound this by applying different cut-offs, none justified with PDAC-specific data [11,12,16].

What the prevalence literature has not addressed is a more clinically relevant question: Which thresholds actually yield different candidate populations and where, across the range of expression, reclassification occurs? This has direct implications for patient selection. We therefore evaluated CLDN18.2 expression in a real-world PDAC cohort and compared the size of the candidate population in parallel according to the thresholds used in different clinical trials.

## 2. Materials and Methods

### 2.1. Study Design and Patients

**Figure 1:**
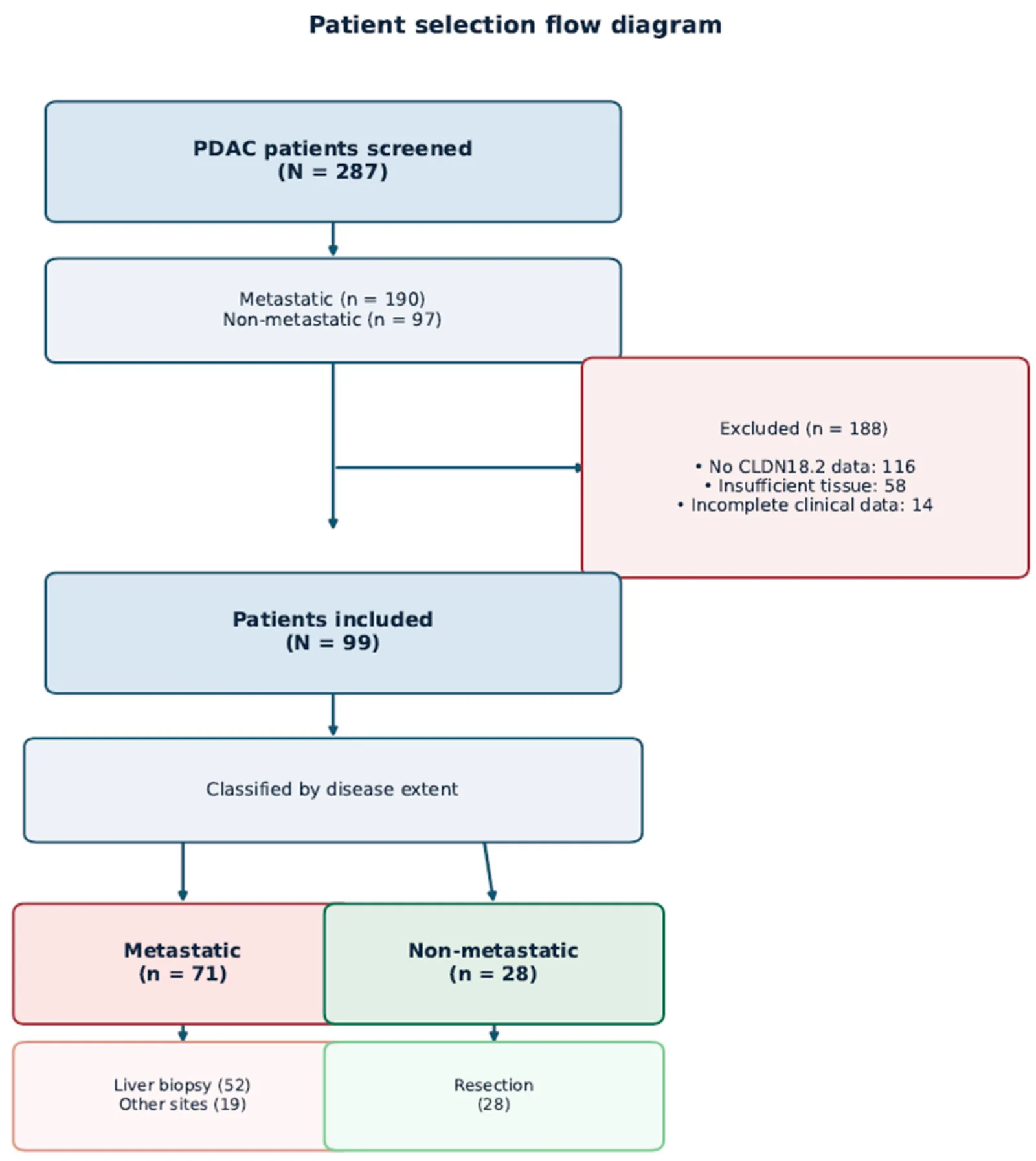
Patient selection flow diagram.

This retrospective, single-center study included patients with histologically or cytologically confirmed PDAC followed at the Department of Medical Oncology, Ankara Etlik City Hospital, between November 2022 and November 2025. It was conducted in accordance with the Declaration of Helsinki and approved by the institutional ethics committee (approval no. AEŞH-BADEK1-2025-710; approval date 24 December 2025). Inclusion required confirmed PDAC, tumor tissue adequate for CLDN18.2 assessment, and sufficient clinical data. A total of 287 patients with PDAC were screened (metastatic, *n* = 190; non-metastatic, *n* = 97). Of these, 188 were excluded: 116 had not undergone CLDN18.2 immunohistochemical testing, 58 had insufficient tumor tissue for CLDN18.2 assessment, and 14 had incomplete clinical data. The remaining 99 patients met the eligibility criteria and were classified according to disease extent at diagnosis as metastatic (*n* = 71) or non-metastatic (*n* = 28). The patient selection process is summarized in Figure 1. In metastatic patients, CLDN18.2 was assessed on pre-treatment diagnostic core biopsies, most frequently from a liver metastasis (52/71, 73.2%) or from other sites (19/71, 26.8%). All non-metastatic patients were assessed on surgical resection specimens of the pancreatic primary (28/28) (Table S1). None of the non-metastatic patients had received neoadjuvant therapy prior to resection, and in metastatic patients CLDN18.2 was assessed on pre-treatment diagnostic biopsies; thus, all specimens reflected treatment-naïve tumor tissue. The following clinicopathological variables were collected: age, sex, tumor grade, lymphovascular invasion, perineural invasion, tumor location, Eastern Cooperative Oncology Group performance status (ECOG PS), serum carbohydrate antigen 19-9 (CA19-9), carcinoembryonic antigen (CEA), albumin, and hemoglobin levels. KRAS mutation status was assessed and categorized as mutant or wild type. In patients with metastatic disease, the presence of liver and peritoneal metastases was additionally recorded.

### 2.2. Immunohistochemistry and Scoring

CLDN18.2 was evaluated on formalin-fixed, paraffin-embedded sections using the 43-14A clone on the Ventana BenchMark platform (Roche Diagnostics, Basel, Switzerland), the companion diagnostic assay used in the SPOTLIGHT and GLOW trials [9,10] and in a PDAC concordance study [17]. Sections were evaluated by two pathologists together, blinded to clinical outcomes, who assigned a single agreed score for each case. Membranous staining intensity (0, 1+, 2+, or 3+) and the percentage of tumor cells at each staining intensity were recorded in 5% increments. Candidate status was subsequently determined according to three predefined thresholds: ≥5% of tumor cells with any membranous staining (1+/2+/3+), ≥40% of tumor cells with any membranous staining (1+/2+/3+), and ≥75% of tumor cells with moderate-to-strong membranous staining (2+/3+). Although the 43-14A antibody recognizes both CLDN18.1 and CLDN18.2 isoforms, the absence of CLDN18.1 expression in pancreatic tissue allows immunohistochemical staining to be interpreted as specific for CLDN18.2.

### 2.3. Thresholds

Two of the three predefined thresholds reflect cut-offs used in current CLDN18.2-directed drug programs, whereas the third was included as an exploratory descriptive bound. The ≥75% moderate-to-strong (2+/3+) threshold corresponds to the zolbetuximab companion diagnostic used in the SPOTLIGHT and GLOW trials [9,10]. The ≥40% threshold (any intensity, 1+/2+/3+) corresponds to the enrollment criterion of the IBI343 antibody–drug conjugate trial in PDAC [11]. The ≥5% threshold, representing the lowest detectable level of expression given that staining was scored in 5% increments, was included as an exploratory lower bound approximating any detectable expression.

### 2.4. Statistical Analysis

Continuous variables are summarized as median (IQR) and compared with the Mann–Whitney U or Kruskal–Wallis test; categorical variables with the Fisher exact or chi-square test. Given the non-normal, bounded distribution of the staining percentage, rank-based tests were used throughout, and no transformation was applied. Eligibility proportions carry Wilson 95% confidence intervals. Because the three thresholds were applied to the same patients, eligibility across thresholds was first compared using Cochran’s Q test, followed by pairwise exact McNemar tests to localize between-threshold reclassification; reclassification rates were reported with 95% confidence intervals. Median follow-up was estimated using the reverse Kaplan–Meier method. Survival was estimated by Kaplan–Meier with log-rank tests, and hazard ratios by univariable Cox regression. Because the survival analysis was not powered for small effects, we estimated the minimum hazard ratio detectable at 80% power for the observed number of events rather than reporting post hoc power. Clinicopathological and survival analyses were prespecified as exploratory; clinicopathological comparisons were additionally assessed with Benjamini–Hochberg correction for multiple comparisons. All statistical analyses were performed in Python (version 3.12.3); two-sided *p* < 0.05 was considered significant.

## 3. Results

### 3.1. Cohort

Of 99 patients, 71 (71.7%) were metastatic and 28 (28.3%) non-metastatic. Baseline characteristics by CLDN18.2 status are shown in Table 1.

### 3.2. CLDN18.2 Positivity Varies According to the Threshold

**Figure 2:**
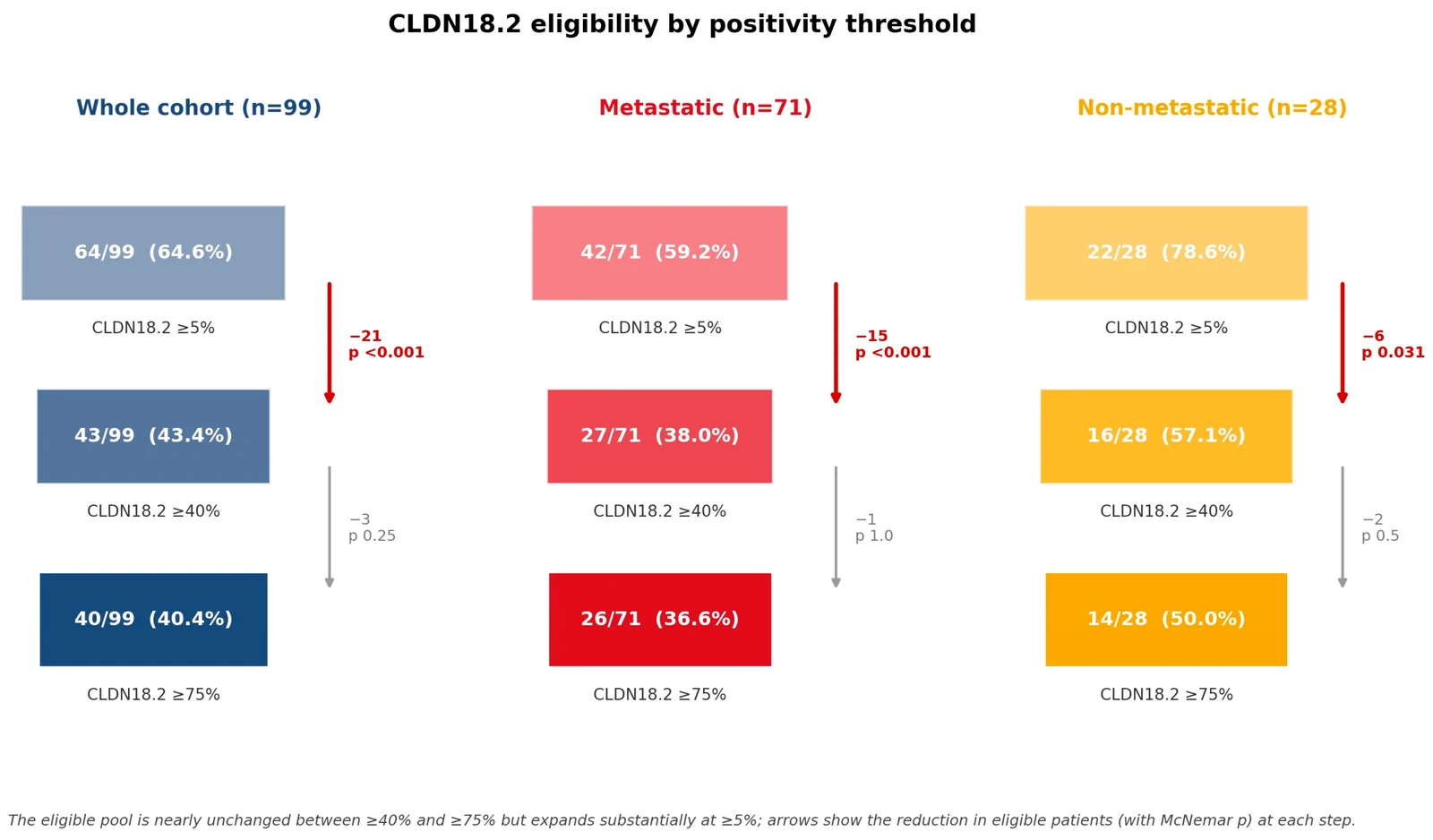
CLDN18.2 candidate proportions at three positivity thresholds in the whole (*n* = 99), metastatic (*n* = 71), and non-metastatic (*n* = 28) cohorts. Bars show candidate patients (number and proportion); arrows between consecutive thresholds indicate the number of patients reclassified as the threshold is changed.

CLDN18.2 positivity at the two clinically applied thresholds was 40.4% (40/99) at ≥75% and 43.4% (43/99) at ≥40%; at the exploratory ≥5% bound (any detectable expression), positivity was 64.6% (64/99). The metastatic subgroup showed the same pattern (36.6%, 38.0%, and 59.2%, respectively), as did the non-metastatic subgroup at higher absolute rates (50.0%, 57.1%, and 78.6%) (Table 2, Figure 2). The higher positivity in the non-metastatic subgroup should be interpreted in light of the different specimen types (resection vs. biopsy).

The candidate proportion differed significantly across the three thresholds in all cohorts (Cochran Q *p* < 0.001 for the whole and metastatic cohorts, *p* = 0.0015 for the non-metastatic subgroup). Pairwise comparisons localized this difference consistently: tightening from ≥40% to ≥75% reclassified almost no one (McNemar *p* = 0.25 overall, *p* = 1.0 metastatic, *p* = 0.5 non-metastatic), whereas relaxing to ≥5% reclassified substantially more patients (24 overall, *p* < 0.001; 16 metastatic, 22.5% [95% CI 14.4–33.5], *p* < 0.001; 8 non-metastatic, *p* = 0.008).

The impact of threshold selection on patient classification was strongly asymmetric. Tightening the threshold from ≥40% to ≥75% resulted in virtually no reclassification, affecting only one metastatic patient (1.4%; McNemar *p* = 1.0) and three patients overall (3.0%). In contrast, lowering the threshold from ≥40% to ≥5% reclassified 15 metastatic patients (21.1%), while lowering it from ≥75% to ≥5% reclassified 16 metastatic patients (22.5%; 95% CI, 14.4–33.5; *p* < 0.001) from negative to positive. Overall, the ≥40% and ≥75% thresholds identified largely overlapping patient populations, whereas application of the ≥5% threshold substantially expanded the CLDN18.2-positive population.

### 3.3. CLDN18.2 Expression Distribution

Among tumors with any CLDN18.2 expression (≥5%), the median staining percentage was high and identical in the metastatic and non-metastatic cohorts (80%). Expression was distributed towards the extremes of the range: only 3 of 99 patients in the whole cohort (and a single metastatic patient) had staining in the 40–74% range, whereas 40 patients (40.4%) had staining in ≥75% of tumor cells and 21 (21.2%) had low-level expression (5–39%). This scarcity of tumors in the 40–74% range explains why the ≥40% and ≥75% thresholds identified nearly identical candidate populations, with reclassification occurring almost entirely at the lower end of the range.

### 3.4. Clinicopathological Associations

After Benjamini–Hochberg correction, none of these exploratory associations remained significant (Table 1). Before correction, CLDN18.2-positive tumors were more frequently well or moderately differentiated (*p* = 0.009); nominal associations were also observed with older age, less frequent liver metastasis, tumor location, and perineural invasion in the non-metastatic subgroup, none of which survived correction. No associations were observed with sex, ECOG performance status, smoking, KRAS mutation status, lymphovascular invasion, CA19-9, CEA, albumin, or hemoglobin.

### 3.5. Association Between CLDN18.2 Expression and Survival

The survival analysis was underpowered: with 52 deaths, it was powered to detect only a hazard ratio of 2.24 or greater, and the following null findings should therefore be interpreted with caution. At a median follow-up of 21.6 months in the metastatic cohort, CLDN18.2 expression was not associated with overall survival (OS) or progression-free survival (PFS) at any positivity threshold (Table 3). At the ≥75% threshold, median OS was 10.3 versus 10.6 months (HR 0.83, 95% CI 0.46–1.49; *p* = 0.530), whereas at the ≥5% threshold, median OS was 10.7 versus 7.9 months (HR 0.76, 95% CI 0.44–1.32; *p* = 0.328). CLDN18.2 expression analyzed as a continuous variable was not associated with OS (HR 1.01 per 10% increase; *p* = 0.893). PFS results were similarly null across all thresholds (all *p* > 0.5; Table 3).

**Table 4:** Univariable and multivariable Cox regression for overall and progression-free survival in the metastatic cohort.

Variable	Univariable		Multivariable	
	HR (95% CI)	*p*	HR (95% CI)	*p*
Overall Survival				
CLDN18.2 (per 10% increment)	0.98 (0.91–1.05)	0.502	0.96 (0.90–1.04)	0.312
Log CA19-9	1.14 (1.02–1.28)	0.023	1.16 (1.02–1.31)	0.021
Liver metastasis	2.05 (0.86–4.85)	0.104	1.72 (0.71–4.16)	0.230
Peritoneal metastasis	0.94 (0.52–1.69)	0.823		
Progression-Free Survival				
CLDN18.2 (per 10% increment)	0.99 (0.94–1.06)	0.842	0.99 (0.93–1.05)	0.689
Log CA19-9	1.14 (1.03–1.27)	0.011	1.15 (1.03–1.28)	0.013
Liver metastasis	1.90 (0.90–4.04)	0.094	1.61 (0.75–3.49)	0.225
Peritoneal metastasis	1.10 (0.65–1.87)	0.729		

## 4. Discussion

Although the development of CLDN18.2-targeted therapies for PDAC is ongoing, the optimal CLDN18.2 expression threshold for defining the candidate population has not yet been established. Two observations regarding CLDN18.2 expression in PDAC are well established: approximately half of tumors express CLDN18.2, and reported positivity rates vary substantially across studies. The present study extends these observations by showing that, within a single cohort assessed using a validated assay, the two thresholds currently used in therapeutic development (≥40% and ≥75%) identified nearly identical candidate populations, whereas lowering the threshold to any detectable expression increased the candidate pool by more than half. This distinction has direct practical implications, because the choice of threshold directly determines the screen-failure rate and the population to which a trial’s results will apply.

Previous studies have reported considerable variability in CLDN18.2 positivity across PDAC cohorts. In the original work identifying CLDN18.2 as a target in pancreatic neoplasms, Wöll et al. reported CLDN18.2 positivity in 59.2% of primary PDAC samples, the majority (54.6%) with moderate-to-strong (≥2+) intensity; metastases of pancreatic neoplasms were also frequently positive (69.4% of lymph node and 65.7% of liver metastases) [8]. Valentini et al. assessed CLDN18.2 in 70 PDAC specimens (28 resections and 42 fine-needle aspiration biopsies) and reported positivity in 50%, with higher expression associated with well- and moderately differentiated tumors and lymph node-negative status [18]. Park et al., in 130 resected PDACs scored as ≥80% of tumor cells with 2+/3+ intensity, found positivity in only 31.5%, associated with well-differentiated histology (*p* < 0.001) and less regional node involvement (*p* = 0.045) [19], and a large digestive-tract series reported 38% (28/74) of PDACs positive at the ≥75% moderate-to-strong threshold [20]. Using the clinically validated 43-14A companion clone with a ≥75%/≥2+ definition, Arseneau et al. found 39 of 120 PDACs (32.5%) positive, with a significant association between lower tumor grade and higher 43-14A staining (*p* < 0.05) [21]. When these disparate cohorts are pooled, a recent systematic review and meta-analysis of 16 studies and 2025 patients estimated a prevalence of 51.6%, but with extreme heterogeneity (I^2^ = 95.4%) that was attributable largely to differences in antibody clone and positivity threshold rather than to geography [22]. An individual-patient-data meta-analysis of six studies (972 patients) similarly placed pooled positivity at 43.5% [23]. In our own cohort, the candidate proportion likewise depended heavily on the definition applied, ranging from 40.4% at the strictest threshold (≥75%) to 64.6% at the lowest (≥5%), figures that sit squarely within this reported range. This wide variation carries a practical implication: because a substantial proportion of PDACs express CLDN18.2 at levels below the ≥75% gastric-derived cut-off, lowering the target threshold for CLDN18.2-directed trials could considerably enlarge the pool of candidate patients—a consideration of particular importance in a disease with few therapeutic options.

That line—the positivity threshold—has never been defined for PDAC and has instead been inherited from gastric cancer. The ≥75% moderate-to-strong standard was established in the phase 3 SPOTLIGHT and GLOW trials of zolbetuximab, building on the earlier phase 2b FAST trial, which had demonstrated a survival benefit in tumors with moderate-to-strong staining in >70% of cells but not in those with 40–69% expression [9,10,24]. Across SPOTLIGHT and GLOW, the prevalence of positivity at this ≥75% cut-off in gastric/gastroesophageal adenocarcinoma was 38.4% [10]. Antibody–drug conjugates (ADCs), however, exploit a bystander effect that can act at lower antigen density, and their programs have consequently adopted lower and inconsistent cut-offs: the anti-CLDN18.2 ADC IBI343 enrolled advanced PDAC patients at a ≥40% threshold (≥2+ intensity) and analyzed efficacy by ≥60% versus <60%, with benefit most pronounced at ≥60% [11]; LM-302 uses ≥50% with 2+/3+ intensity [25]; and the afucosylated antibody FG-M108 has enrolled first-line PDAC patients at a threshold as low as ≥10% [16]. Beyond ADCs, the anti-CLDN18.2/CD3 bispecific antibody IBI389 similarly enrolled PDAC patients at ≥10% (2+/3+) [12]. Emerging modalities such as CAR-T cells and cell-engaging or sensitization-based approaches—including sono-immunotherapy strategies under preclinical development in pancreatic cancer [26]—are likely to shift these requirements further, since the antigen density needed for efficacy varies with the therapeutic mechanism. In other words, the “positive” population in PDAC is defined differently by nearly every drug program, none of them validated in PDAC itself. Our choice of ≥5% as the lowest threshold was pragmatic rather than arbitrary: staining was scored in 5% increments, so ≥5% represents the lowest detectable expression—the operational equivalent of “any expression.” Reporting candidate proportions across this full span is clinically consequential, because CLDN18.2-directed therapy remains investigational in PDAC.

In a setting where effective options are scarce, and enrollment into biomarker-selected trials is a practical bottleneck, the size of the candidate pool is not a trivial matter. Our data show that this pool can be enlarged substantially—from roughly four in ten patients at ≥75% to nearly two-thirds at ≥5%—simply by relaxing the threshold, an expansion that could meaningfully accelerate trial accrual. Early efficacy data lend cautious support to a lower bar: FG-M108 combined with gemcitabine and nab-paclitaxel produced an objective response rate of 50% in untreated PDAC selected at ≥10% expression [16], and IBI343 in the ≥60% subgroup achieved a PDAC response rate of 40% [11]. The precedent of trastuzumab deruxtecan is instructive here: by demonstrating clinical benefit in HER2-low breast cancer—a population previously considered ineligible for HER2-directed therapy—it showed that antibody–drug conjugates can extend benefit to tumors with lower target expression, reframing the biomarker threshold from a strict binary into a broader eligibility continuum [27]. By analogy, a lower threshold might expand the candidate pool for CLDN18.2-directed ADCs in PDAC without forfeiting benefit—but this remains hypothetical, since our data speak only to candidate numbers, not to treatment response. However, this enthusiasm must be tempered by the same trials’ evidence that the benefit is expression-dependent: in the IBI343 dose-expansion cohort, CLDN18.2-negative patients had no objective responses and markedly shorter progression-free survival than positive patients (HR 0.198) [11]. Lowering the threshold, therefore, widens the pool but risks diluting the benefit, and the appropriate cut-off can ultimately be settled only by response-anchored, PDAC-specific data—which our study underscores the need for but cannot itself provide. Because no patient in our cohort received CLDN18.2-directed therapy, our analysis quantifies how thresholds reshape the candidate population but cannot determine which threshold best predicts treatment response; any suggestion that a lower threshold might be clinically appropriate, therefore, remains a hypothesis to be tested prospectively rather than a conclusion supported by our data. Conceptually, an expression-based threshold indicates only how many patients carry the target, whereas a response-anchored threshold would define the expression level at which clinical benefit actually begins—the distinction that ultimately matters for selecting patients.

Beyond candidate selection, we examined the clinicopathological associations of CLDN18.2, and our findings were largely null, with two exceptions that align with the literature. CLDN18.2-positive tumors in our cohort were more often well differentiated and, among metastatic patients, less frequently associated with liver metastasis. The relationship with differentiation is one of the most consistent in this field: Park et al. found positivity strongly associated with well-differentiated histology (*p* < 0.001) [19]; Valentini et al. reported higher expression in well- and moderately differentiated tumors [18]; and Cha et al., in 95 resected PDACs, found high CLDN18.2 expression (21.1%) associated with better differentiation (*p* = 0.016), absence of lymphatic invasion (*p* < 0.001), and lower nodal stage (*p* = 0.002) [28], and the meta-analysis confirmed significantly lower expression in poorly differentiated (G3) tumors [22]. Associations with perineural and lymphovascular invasion were, for the most part, statistically non-significant. In the non-metastatic subgroup, however, CLDN18.2-positive tumors more frequently exhibited perineural invasion (*p* = 0.022), an association not observed in the metastatic subgroup or in the cohort as a whole. Given its confinement to a single small subgroup, its direction opposite to our other associations, and the absence of correction for multiple comparisons, this finding is most plausibly incidental. Even within PDAC, an inverse association between CLDN18.2 and vascular invasion has previously been reported, which we were unable to reproduce in our smaller subgroups [19]. Overall, the limited sample size makes it difficult to establish firm associations between CLDN18.2 expression and clinicopathological features in our cohort.

Our survival findings warrant particular attention because they diverge from part of the literature. In our metastatic cohort, CLDN18.2 was not significantly associated with overall or progression-free survival at any threshold. Although a numerical trend toward longer survival was observed in expressing tumors at the lowest cut-off (median OS 10.7 vs. 7.9 months at ≥5%), this did not reach statistical significance and was not seen at higher thresholds; expression also showed no dose-dependent effect among positive tumors. This is fully concordant with Park et al., who found no survival difference between positive and negative resected PDACs (17.4 vs. 20.6 months, *p* = 0.770) [19]. In contrast, however, several reports have reported a favorable prognostic effect: the individual-patient-data meta-analysis found improved overall survival with positivity, most pronounced in Asian and resected populations (pooled HR 0.74) [23], and Arseneau et al. reported better survival in CLDN18.2-positive cases at the cohort’s median overall survival of 23 months [21]. Several factors likely reconcile this discrepancy. Most positive reports derive from resected, earlier-stage, and predominantly Asian cohorts, whereas ours is a real-world, biopsy-based, predominantly metastatic population. Moreover, our survival analysis was underpowered: with 52 deaths, we had 80% power to detect only a hazard ratio of 2.24 or greater, so a modest prognostic effect cannot be excluded. Taken together, the balance of evidence—and our own null result across the full range of expression—supports interpreting CLDN18.2 in PDAC primarily as a therapeutic target rather than as a robust prognostic biomarker, consistent with trial data in which expression predicts response to CLDN18.2-directed agents but does not track natural history prognosis [11].

A distinctive feature of our cohort is that, in metastatic patients, CLDN18.2 was read from the tissue clinicians actually obtain—most often a diagnostic liver biopsy—rather than from resection specimens, which supply most published data. This distinction matters. In the concordance study by Kyuno et al., biopsy–resection agreement at ≥75% appeared high (92.5%) but was inflated by the preponderance of negative cases, and the sensitivity of biopsy for detecting a positive tumor was only 54.6%; lowering the biopsy cut-off to 20% was required to reach full sensitivity [17]. This underscores the importance of adequate tissue sampling for reliable molecular and immunohistochemical assessment, particularly for markers evaluated on limited biopsy material; optimizing endoscopic ultrasound–guided tissue acquisition and confirming specimen adequacy help ensure sufficient, representative tissue for such analyses [29,30]. This limitation is compounded by the pronounced intratumoral heterogeneity of PDAC, which spatial single-cell profiling has shown to occur at the level of individual glands in both primary and metastatic tumors, including for classical-subtype markers such as CLDN18.2 [31]. Critically, expression tends to be lower at metastatic sites: in matched primary–liver-metastasis pairs from treatment-naïve metastatic PDAC, CLDN18 was expressed significantly more in the primary tumor than in the liver metastasis (*p* = 0.03) [32]—precisely the setting in which our metastatic cohort was assessed. A biopsy-based candidate estimate may therefore understate the treatable population, a caveat that applies to our metastatic figures and, more importantly, to real-world trial screening, where most patients with unresectable disease are selected on limited biopsy tissue. Although heterogeneity between biopsy and resection specimens may have affected the measured CLDN18.2 staining percentage, this is in line with the known challenge of evaluating membranous immunotherapy biomarkers on limited biopsy material in PDAC, where pronounced intratumoral heterogeneity and the need to pre-screen for low-prevalence targets are well recognized [33]. Combining immunohistochemistry with complementary molecular techniques may help mitigate such tissue-related variability, as increasingly applied to target characterization in gastrointestinal malignancies [34]. Given the different specimen types (primary vs. metastatic tissue), as well as the difference between resection material and the adequacy of biopsy sampling, tissue-related characteristics may have contributed to the difference in expression percentages between the metastatic and non-metastatic groups. Although metastatic biopsies are generally considered adequate for diagnosis and treatment decisions in solid tumors, the reduced sensitivity of biopsy for detecting CLDN18.2 positivity suggests that this specific marker may warrant particular attention.

The therapeutic relevance of CLDN18.2 in PDAC may extend beyond metastatic disease into the curative-intent setting. CLDN18.2 positivity has been consistently reported in resected PDAC, at rates of approximately 20–30% across surgical series [15,19,28], supporting the existence of a potentially targetable population among surgical patients. Its assessment, however, is sensitive to specimen type: in matched samples, concordance between preoperative biopsy and resection was high at the ≥75% threshold (92.5%), yet the sensitivity of biopsy for identifying positive tumors was only 54.6% [17], indicating that preoperative sampling alone may miss eligible patients. Expression may also differ by tumor site; in a large multicenter series (599 primary tumors and 197 lymph nodes), CLDN18.2 was positive in 23% of primary tumors and 17.8% of lymph nodes, with occasional discordance between primary and nodal disease [35].

A related question is whether systemic therapy alters CLDN18.2 expression and thus eligibility for subsequent targeted treatment. In the PANCALYZE cohort, only 1 of 15 neoadjuvant-treated patients was CLDN18.2-positive, compared with 93 of 294 untreated patients (*p* = 0.040), although the small treated subgroup precludes any causal inference [15]. Preliminary translational data likewise suggest that chemotherapy may dynamically modulate cell-surface CLDN18.2 in a treatment- and sensitivity-dependent manner [36]. Whether the marker should therefore be assessed before neoadjuvant therapy, afterwards, or at both time points remains to be defined.

These questions have already entered prospective investigation. The phase Ib study NCT07024615 is evaluating the CLDN18.2/CD3 bispecific T-cell engager ASP2138 as neoadjuvant monotherapy before surgery in resectable disease, followed by adjuvant chemotherapy [37], while the phase II study NCT07415525 is evaluating the antibody–drug conjugate IBI343 before surgery in borderline-resectable disease [13]. Although efficacy data are not yet available, these trials reinforce that the positivity threshold will shape not only which patients are selected but also how and when CLDN18.2 is assessed around surgery.

## 5. Limitations

This study has several limitations. It was a single-center, retrospective analysis of a modest cohort, and the survival analysis was underpowered, with 80% power to detect only a hazard ratio of 2.24 or greater. A further limitation is the potential for selection bias: of 287 screened patients, 188 (65.5%) were excluded, most often because CLDN18.2 testing had not been performed or tissue was inadequate. The evaluable cohort may therefore not fully represent the broader PDAC population, particularly patients in whom tissue acquisition is difficult—for example, those with limited or poorly accessible tumor tissue—and this may limit the generalizability of our findings. CLDN18.2 was scored in 5% increments, which may not capture finer gradations of expression. Because metastatic patients were evaluated on biopsy material—most often from liver metastases—whereas non-metastatic patients were evaluated on resection specimens, disease stage was inseparable from specimen type and site; the difference in expression between these groups is, therefore, reported descriptively and not interpreted biologically. We did not have paired primary and metastatic tissue from the same patients, precluding direct assessment of site-related differences in expression. Beyond these considerations, our threshold-based estimates may not translate directly to other settings, because CLDN18.2 immunohistochemistry is subject to assay-related variability. Reported positivity rates differ markedly across studies, largely reflecting differences in antibody clone and scoring threshold rather than geography. Even with the clinically validated 43-14A companion diagnostic, staining and interpretation can vary across laboratories and platforms, so the same tumor could fall on different sides of a given threshold depending on the assay used. This analytical variability, compounded by differences in positivity rates across geographic and ethnic populations, limits the global generalizability of any single threshold and reinforces the need for standardized, assay-aware threshold definition. Finally, because none of the patients had received CLDN18.2-directed therapy, our candidate-population estimates could not be anchored to treatment response; the thresholds we examined, therefore, describe the size of candidate populations rather than the cut-off that best predicts benefit.

## 6. Conclusions

In a real-world PDAC cohort assessed with the clinical companion diagnostic, the two thresholds currently used in drug development—the intermediate and zolbetuximab cut-offs—selected nearly the same patients, whereas relaxation to any detectable expression enlarged the candidate pool by more than half. CLDN18.2 behaved primarily as a targetable rather than a prognostic marker. Against a literature in which the same disease is called positive at widely different rates and against trial thresholds imported from gastric cancer without disease-specific validation, our findings make a narrow but concrete case: the threshold that will decide who receives these drugs in PDAC cannot be borrowed and must be established prospectively, anchored to treatment response. Prospective studies correlating CLDN18.2 expression with response to CLDN18.2-directed agents in PDAC—ideally using paired primary and metastatic tissue—will be needed to define this threshold.

## Figures and Tables

**Table 1:** Baseline characteristics by CLDN18.2 status (≥5%).

Variable	CLDN18.2 Positive (≥5%) (*n* = 64)	CLDN18.2 Negative (<5%) (*n* = 35)	*p* * (Metastatic)	*p* * (Non-Metastatic)
Age, y, median (IQR)	66 (63–70)	62 (59–68)	0.148	0.273
CA19-9, U/mL, median (IQR)	216 (34–812)	223 (17–1806)	0.848	0.801
CEA, ng/mL, median (IQR)	4 (2–10)	6 (3–25)	0.620	0.435
Albumin, g/dL, median (IQR)	4 (3–4)	4 (3–4)	0.725	0.715
Hemoglobin, g/dL, median (IQR)	12 (11–13)	12 (11–12)	0.566	0.287
Sex, *n* (%)			0.811	0.653
Female	31 (48%)	20 (57%)		
Male	33 (52%)	15 (43%)		
ECOG PS, *n* (%)			0.806	1.000
ECOG 1–2	45 (70%)	24 (69%)		
ECOG 2–3	19 (30%)	11 (31%)		
Smoking, *n* (%)			0.629	0.655
Never	32 (50%)	19 (54%)		
Ex/current	32 (50%)	16 (46%)		
Pathologic stage, *n* (%)			0.099	0.958
Stage 1	6 (9%)	2 (6%)		
Stage 2	18 (28%)	3 (9%)		
Stage 3	10 (16%)	5 (14%)		
Stage 4	30 (47%)	25 (71%)		
Tumor location, *n* (%)			0.047	0.742
Head	33 (52%)	22 (63%)		
Body	4 (6%)	4 (11%)		
Tail	13 (21%)	2 (6%)		
Mixed	13 (21%)	7 (20%)		
KRAS (tested only), *n* (%)			0.613	0.115
Wild-type	5 (12%)	4 (31%)		
Mutant	37 (88%)	9 (69%)		
LVI, *n* (%)			1.000	0.653
No	21 (52%)	7 (47%)		
Yes	19 (48%)	8 (53%)		
PNI, *n* (%)			1.000	0.022
No	11 (28%)	8 (53%)		
Yes	29 (72%)	7 (47%)		
Differentiation, *n* (%)			0.259	0.009
Well	12 (29%)	5 (28%)		
Moderate	28 (67%)	7 (39%)		
Poor	2 (5%)	6 (33%)		
Liver metastasis, *n* (%)			0.039	-
No	10 (26%)	1 (4%)		
Yes	28 (74%)	24 (96%)		
Peritoneal metastasis, *n* (%)			0.198	-
No	19 (50%)	17 (68%)		
Yes	19 (50%)	8 (32%)		
Metastasis timing, *n* (%)			0.116	-
de novo	21 (68%)	21 (88%)		
metachronous	10 (32%)	3 (12%)		

* Each *p*-value compares CLDN18.2-positive versus CLDN18.2-negative patients within the respective cohort (metastatic or non-metastatic). Percentages are calculated from patients with available data; data were incomplete for several variables (KRAS, lymphovascular invasion, perineural invasion, and metastasis timing, *n* = 55; differentiation, *n* = 60; liver and peritoneal metastasis, *n* = 63; CEA, *n* = 95). Categorical variables were compared using the Fisher exact or chi-square test, and continuous variables were compared using the Mann–Whitney U test. *p*-values are unadjusted for multiple comparisons; no association remained significant after the Benjamini–Hochberg correction. CEA, carcinoembryonic antigen; ECOG PS, Eastern Cooperative Oncology Group Performance Status; IQR, interquartile range; LVI, lymphovascular invasion; PNI, perineural invasion.

**Table 2:** CLDN18.2 candidate proportions and between-threshold reclassification.

Cohort	≥5% *n*/*N* (%)	≥40% *n*/*N* (%)	≥75% *n*/*N* (%)	Reclassified ≥75% → ≥5% *n* (%) [95% CI]	McNemar *p*
Whole (*n* = 99)	64/99 (64.6)	43/99 (43.4)	40/99 (40.4)	24 (24.2) [16.9–33.5]	<0.001
Metastatic (*n* = 71)	42/71 (59.2)	27/71 (38.0)	26/71 (36.6)	16 (22.5) [14.4–33.5]	<0.001
Non-metastatic (*n* = 28)	22/28 (78.6)	16/28 (57.1)	14/28 (50.0)	8 (28.6) [15.3–47.1]	0.008

**Table 3:** CLDN18.2 and survival in the metastatic cohort (*n* = 71).

Comparison	Endpoint	*n* (Higher)	Median, Higher (mo)	*n* (Lower)	Median, Lower (mo)	HR (95% CI)	Log-Rank *p*
≥5% vs. negative	OS	42	10.7	29	7.9	0.76 (0.44–1.32)	0.328
	PFS	42	6.4	29	4.2	0.86 (0.52–1.43)	0.571
≥40% vs. <40%	OS	27	10.3	44	9.3	0.80 (0.45–1.43)	0.452
	PFS	27	6.5	44	4.3	0.96 (0.57–1.60)	0.879
≥75% vs. <75%	OS	26	10.3	45	10.6	0.83 (0.46–1.49)	0.530
	PFS	26	6.5	45	5.2	0.94 (0.56–1.58)	0.824
Within pos: ≥75% vs. 5–74%	OS	26	10.3	16	10.9	1.01 (0.47–2.17)	0.976
	PFS	26	6.5	16	5.7	1.09 (0.55–2.14)	0.798

Univariable and multivariable Cox regression were performed in the metastatic cohort (62 patients with complete data; 46 deaths, 57 progression events) (Table 4). CLDN18.2, log-transformed CA19-9, and liver and peritoneal metastases were tested univariably; the multivariable model retained CLDN18.2 together with log-CA19-9 and liver metastasis. After adjustment, CLDN18.2 was not independently associated with OS (HR 0.96 per 10% increment, 95% CI 0.90–1.04; *p* = 0.312) or PFS (HR 0.99, 95% CI 0.93–1.05; *p* = 0.689), whereas log-CA19-9 remained prognostic (OS *p* = 0.021; PFS *p* = 0.013).

## Data Availability

The data presented in this study are available on reasonable request from the corresponding author. The data are not publicly available due to patient privacy restrictions.

## Supplementary Materials

The following supporting information can be downloaded at https://www.mdpi.com/article/10.3390/cancers18172827/s1. Table S1: Specimen type and anatomical site by cohort.

## Abbreviations

The following abbreviations are used in this manuscript: CLDN18.2, claudin 18.2; PDAC, pancreatic ductal adenocarcinoma; IHC, immunohistochemistry; OS, overall survival; PFS, progression-free survival; HR, hazard ratio; CI, confidence interval; ADC, antibody–drug conjugate; ECOG PS, Eastern Cooperative Oncology Group performance status; CA19-9, carbohydrate antigen 19-9; CEA, carcinoembryonic antigen; IQR, interquartile range.
