# Supplementary material for: Claudin 18.2 Positivity Thresholds and Candidate Populations for Targeted Therapy in Pancreatic Ductal Adenocarcinoma: A Real-World Retrospective Cohort
Source: Cancers (Basel). 2026 Sep 1;18(17):2827. doi: 10.3390/cancers18172827 (PMC13565671; doi:10.3390/cancers18172827)
Supplement: Supplementary file 1 [file cancers-18-02827-s001.zip › cancers-4519612-supplementary.pdf]

Supplementary Table S1. Specimen type and anatomical site by cohort

Cohort	Specimen type / site	n (%)
**Metastatic (n=71)**	**Diagnostic core biopsy**	
	Liver metastasis	52 (73.2%)
	Other/unspecified (primary or peritoneum)	19 (26.8%)
**Non-metastatic (n=28)**	**Surgical resection**	
	Resected pancreatic primary	28 (100%)
